# Histological tumor response predicts clinical outcome in patients with colorectal peritoneal metastasis treated with preoperative chemotherapy followed by cytoreduction and HIPEC

**DOI:** 10.1515/pp-2022-0117

**Published:** 2023-03-27

**Authors:** Isa Valéria Ferreira de Sousa, Joanne M.D. Lopes, Jorge P.M. Nogueiro, Teresa R. Costa, Laura E.R. Barbosa, Marisa M.M. Aral

**Affiliations:** Faculty of Medicine of Porto University, Porto, Portugal; Anatomic Pathology Department, São João University Hospital Center, Porto, Portugal; General Surgery Department, São João University Hospital Center, Porto, Portugal; General Surgery Department, Local Health Unit of Guarda, Guarda, Portugal

**Keywords:** colorectal cancer, cytoreduction surgery, histological response, hyperthermic intraperitoneal chemotherapy, peritoneal carcinomatosis, peritoneal regression grading score, preoperative chemotherapy, tumor grading, tumor regression

## Abstract

**Objectives:**

Up to one quarter of the patients with colorectal cancer (CRC) develop peritoneal carcinomatosis (PM). The aims of this retrospective study were to characterize the histological response of the PM of CRC to preoperative chemotherapy and evaluate the potential prognostic value, in terms of survival.

**Methods:**

This retrospective unicentric study evaluated a group of 30 patients treated between 2010 and 2020 at the São João University Hospital Center with preoperative chemotherapy, followed by cytoreduction surgery plus hyperthermic intraperitoneal chemotherapy. The evaluation of the histological response was done using two scores: the tumor regression grading (TRG) and the peritoneal regression grading score (PRGS).

**Results:**

Mean post-procedure survival is higher in the PRGS 1–2 group (74.19 months) vs. the PRGS 3–4 group (25.27 months) (p=0.045), as well as in the TRG 1–2 group (74.58 months) vs. TRG 4–5 (25.27 months) (p=0.032). As for progression-free survival (PFS), the PRGS 1–2 group had a mean value of 58.03 months vs. PRGS 3–4 which had 11.67 months (p=0.002). Similar was observed with the TRG 1–2 group, which had a mean PFS of 61.68 months vs. TRG 4–5 with 11.67 months (p=0.003).

**Conclusions:**

A better histological response to preoperative chemotherapy, represented as a lower PRGS and TRG value, is associated with longer post-procedure survival and progression-free survival in this group of patients. That is, these two scores have prognostic value.

## Introduction

Up to one quarter of the patients with colorectal cancer (CRC) develop peritoneal carcinomatosis [1], synchronous or metachronous, and about 8% of patients have it at the time of primary resection [2]. This is associated with a significantly shorter overall and progression-free survival when compared with other manifestations of metastatic CRC [2].

In patients with metastatic disease, the survival has been growing substantially in the last two decades. In the past, peritoneal carcinomatosis was seen as a terminal stage of CRC and the patients were only considered for palliative care. Currently, the treatment of choice in selected patients is preoperative chemotherapy, followed by cytoreduction surgery (CRS) plus hyperthermic intraperitoneal chemotherapy (HIPEC). This treatment may offer these patients a chance for long-term survival and even for a cure [3]. Due the improvement of surgical techniques and chemotherapy modalities, nowadays it is possible to achieve an overall survival of 41 months [1].

Regarding preoperative chemotherapy, there are multiple regimens available for these patients and it is used in selected cases in which a downstaging is needed because of tumor growth or extension into nearby vital structures. This also can identify nonresponders who may not benefit from CRS, it may avoid or limit extraperitoneal spread of the malignancy and it also could reduce the amount of disease, increasing the possibility of a complete CRS [3]. This means that this chemotherapy can convert an unresectable disease in to a resectable one [4].

Later, the cytoreductive surgery aims to resect all the macroscopic disease in the peritoneal cavity [5] as first described by Sugarbaker, and then it’s complemented with hyperthermic intraperitoneal chemotherapy to eliminate the residual microscopic disease.

During the surgical procedure, the peritoneal carcinomatosis index (PCI) is estimated, and it represents the extent of disease in the peritoneal cavity [6]. In the end of the resections, it is obtained the Completeness of the cytoreduction score. Complete resection is considered when we have CC-0 or CC-1 [7].

The survival of these patients also depends on the PCI and the completeness of the cytoreduction, which are independent prognostic factors of survival [5]. The role of the heated intraperitoneal chemoperfusion remains not completely established [8].

The response to preoperative chemotherapy is typically evaluated by radiological methods but those have an important limitation: peritoneal metastasis (PM) less than 1 cm in diameter can be difficult to detect by those exams. For this reason, the histological tumor response assessment has been gaining more relevance and it has been identified and recognized as an important prognostic factor in patients treated with preoperative chemotherapy for CRC metastasis [3]. This evaluation can be done using different scores.

Rubbia-Brandt et al. described in 2007 the tumor regression grade (TRG) for hepatic metastasis of CRC. It is divided in 5 grades: TRG1 – absence of tumor cells and total replacement for fibrosis; TRG2 – rare tumor cells scattered in abundant fibrosis; TRG3 – more tumor cells in a fibrotic area; TRG4 – predominance of tumor cells; TRG5 – exclusively tumor cells [9].

In 2016, the peritoneal regression grading score was described as a new possible histological scoring system for PM, not specifically for CRC. This score has four categories based on the presence of residual tumor cells and the regression characteristics such as fibrosis, infarct-like necrosis, and acellular mucin: PRGS 1 (complete regression with total absence of tumor cells); PRGS 2 (major regression features with only a few tumor cells i.e. major histological response); PRGS3 (few regression features and predominantly tumor cells i.e. minor histological response); PRGS 4 (absence of response to therapy i.e. absence of any regression feature) [1, 9, 10].

Many studies have shown that complete or major histological response after chemotherapy is associated with better survival in patients with digestive cancer. For PM from CRC, there’s a lack of studies that evaluate the prognostic value of PRGS.

Therefore, the aims of this retrospective study were to characterize the histological response of the peritoneal metastasis of colorectal cancer to preoperative chemotherapy and evaluate the potential prognostic value, in terms of overall survival, post-procedure survival and progression-free survival.

## Patients and methods

### Study design and patients

This observational retrospective study evaluated 30 patients treated between 2010 and 2020 at the São João University Hospital Center (SJUHC) with preoperative chemotherapy, followed by cytoreduction surgery plus hyperthermic intraperitoneal chemotherapy.

Seven patients were excluded from an initial cohort of 37 because of: no preoperative chemotherapy (n=5), no CRS due to high PCI (n=1) and lack of clinical information (n=1).

The clinical, surgical and follow-up information was obtained from the hospital data base. The histological specimens obtained from the CRS were recovered from the archive of the Hospital Center and they were observed in the Anatomic Pathology Department.

The inclusion criteria are confirmation of peritoneal carcinomatosis, performed preoperative chemotherapy, realization of CRS followed by HIPEC in the same surgical time.

The characteristics of the study population are listed in Table 1. Of the 30 patients, 17 are female. The mean age at diagnosis of the primary tumor is 52.03 years and the mean age at the time of surgery is 53.87 years.

**Table 1:** Clinical and demographic characteristics.

Clinical and demographic characteristics	n	%
Sex		

Male	13	43.3
Female	17	56.7

Primary tumor		

Right colon	9	30
Left colon	17	56.7
Colon (both)	1	3.3
Rectum	3	10

ECOG		

0	29	96.7
1	1	3.3

Peritoneal carcinomatosis		

Synchronous	16	53.3
Metachronous	14	46.7

TNM		

T2	1	3,3
T3	11	36.7
T4	18	60

Molecular status		

RAS wild-type	10	33.3
RAS mutated	19	63.3
Missing	1	3.3

Death		

Yes	17	56.7
No	13	43.3

Total	30	100

Regarding the location of the primary tumor, 28 corresponded to colon tumors and two to rectal tumors, most of which were in a T4 stage.

### Preoperative chemotherapy

This group of patients differs on the preoperative chemotherapy regimen (Table 2). The most used regimen was the association of FOLFIRI with Bevacizumab, anti-VEGF agent. This chemotherapy was stopped 4 weeks before surgery (or 6 weeks if Bevacizumab), according to the department protocol.

**Table 2:** Preoperative chemotherapy.

Regimens	n	%
FOLFIRI	1	3.3
FOLFIRI + Bevacizumab	9	30.0
FOLFIRI + Cetuximab	4	13.3
FOLFIRI + Panitumumab	1	3.3
FOLFOX	7	23.3
FOLFOX + Bevacizumab	3	10.0
FOLFOX + Panitumumab	1	3.3
5-FU + LV + Bevacizumab	1	3.3
Xeliri	1	3.3
Missing	2	6.7
Total	30	100

### Cytoreduction surgery

The surgical procedure started with a median laparotomy and then the surgeon explores carefully all the peritoneal cavity and does multiple resections as needed. In some cases, it is also necessary to do organ resections, as described by Sugarbaker. The aim is to get a complete cytoreduction, defined by CC-0 or CC-1.

The mean duration of surgery, including HIPEC, was 554.97 min. The mean value of the PCI was 6.63 and a complete resection (CC-0 or 1) was achieved in at least 83.3% of the patients.

### Hyperthermic intraperitoneal chemotherapy

Half of the patients had a 30 min HIPEC with intraperitoneal oxaliplatin in association with intravenous 5-fluorouracil and leucovorin. The remaining half had a 90 min infusion with mitomycin C plus cisplatin (n=14) or mitomycin C only (n=1). The open technique, also known as the coliseum technique, was used in all patients.

### Evaluation of histological response

The histological material of peritoneal metastasis removed during the cytoreduction surgery, fixed in hematoxylin–eosin, were reviewed in the Anatomic Pathology Department of the SJUHC, by the main investigator and by a hospital assistant graduated in Anatomical Pathology, representative of the service in the multidisciplinary oncological meetings (tumors of the colon and rectum; tumors of the liver and pancreas). The pathologist was unaware of clinical information, chemotherapy regimen and patient outcome, that is, this assessment was single-blinded.

The histological characteristics mentioned in the TRG and PRGS scores, such as presence/absence of tumor cells and their approximate percentage, presence and percentage of fibrosis, presence/absence of acellular mucin pools, and presence/absence of ischemic necrosis were evaluated. Other potential features of tumor regression and response to chemotherapy were also evaluated, such as the presence/absence of xanthelasmized macrophages/xantogranulomatous reaction, type of inflammatory infiltrate in the tumor and in healthy tissue, and histological grade. We also evaluated the existence of venous, lymphatic and perineural invasion, as well as lymph node metastasis.

As for the histological type, they were classified as mucinous (with >50% mucinous differentiation) or non-mucinous (<50% mucinous differentiation). As for the histological grade, they were divided into grade 1 (well differentiated), grade 2 (moderately differentiated), grade 3 (poorly differentiated) and grade 4 (undifferentiated).

Each surgical fragment gave rise to multiple blades. Among these, it was considered the worst (in terms of regression) for classification purposes.

Considering what was observed during the observation of the slides, we considered the worst value of TRG and PRGS, that is, the highest value, as well as the mean value.

### Follow-up

After the CRS plus HIPEC procedure, patients were seen in medical appointment every 3 months for 3 years, then every 6 months thereafter. The follow-up also included CT or MRI every 3 months for the first 3 years, then every 6 months for the next 2 years. Serial measurement of tumor markers was also part of the follow-up of these patients.

With the data obtained from the clinical follow-up of the patients, we evaluated their overall survival and the post-procedure survival as well as progression-free survival.

### Statistical analysis

The statistical analysis involved measures of descriptive statistics (absolute and relative frequencies, means, medians and standard deviations) and inferential statistics. In the latter, the Chi-square test and Fisher’s exact test were used, as appropriate.

Overall survival was defined as the time between the date of the primary diagnosis and the death or last follow-up date. Post-procedure survival was defined as the time between the date of the CRS-HIPEC and the death or last follow-up date. Progression-free survival was defined as the time between the date of the procedure and the date of evidence of progression.

Cumulative survival curves for post-procedure survival and progression-free survival were obtained by the Kaplan–Meier method and the log rank test was used to compare differences between groups. Hazard ratio (HR) and respective 95% confidence intervals (CI) were calculated by cox regression model. Logistic regression was used to determine the association between the risk of progression and the respective odds ratios (ORs).

Statistical significance was defined for p-values ≤0.05. Statistical analysis was performed with SPSS^®^ 26.0 for Mac.

## Results

### Histological findings

**Figure 1:**
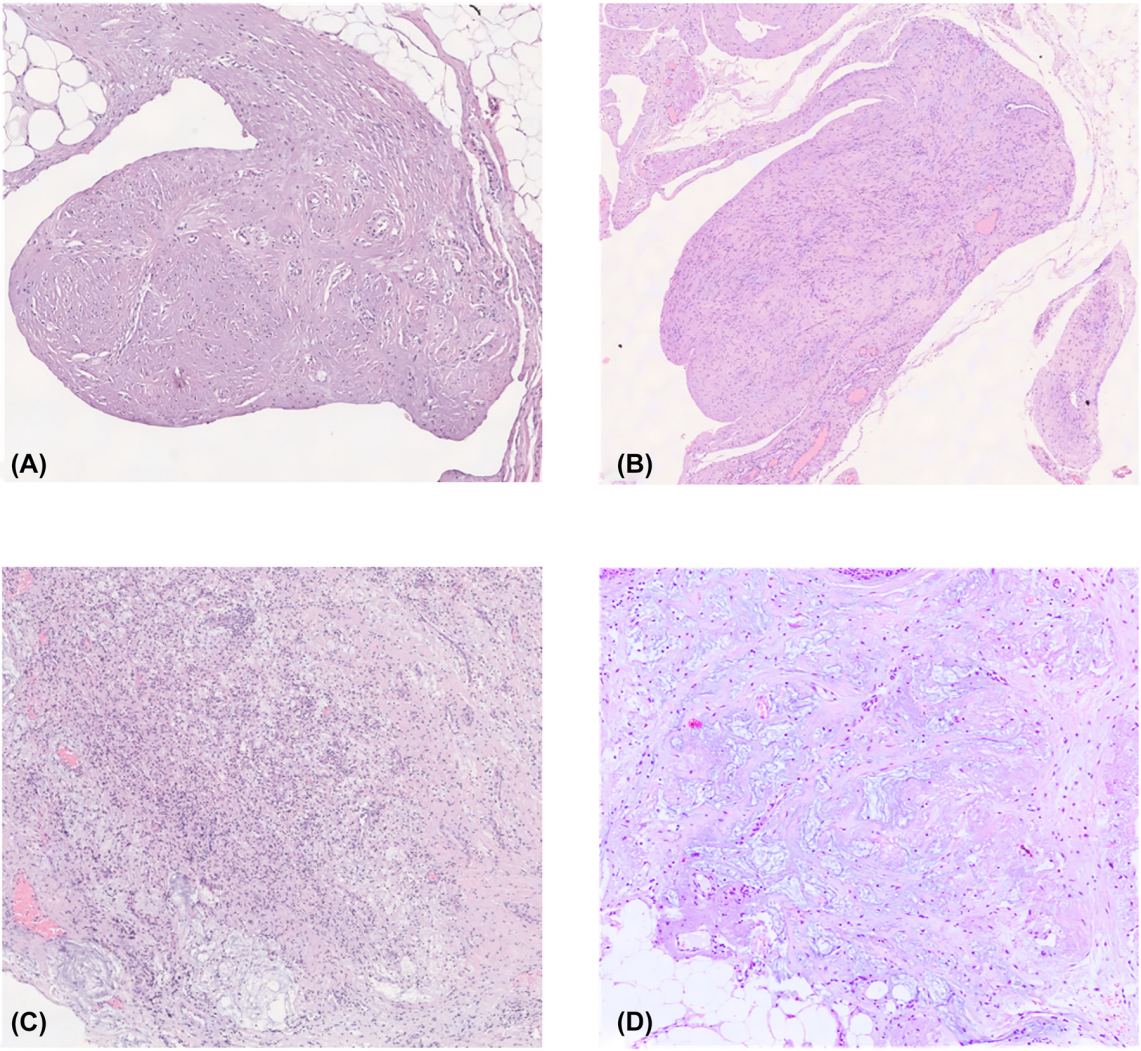
Histological findings of regression. (A) Complete regression; (B) complete regression; (C) foamy macrophages and acellular mucins; and (D) pools of acellular mucins.

The observed characteristics and the classification used are shown in Table 3. The most frequently observed histological type was non-mucinous. The most frequent histological finding was fibrosis, and to a lesser extent pools of acellular mucins, xanthelasmized macrophages/xantogranulomatous reaction, giant cell reaction, lymphatic invasion and dystrophic calcifications. Figure 1 shows some of the observed findings.

**Table 3:** Histological findings (^a^within the specimens with tumor).

Observed characteristic	Classification	n	%
Histological type^a^	Mucinous/Non mucinous	4/11	26.7/73.3
Grade of differentiation^a^	Grade 1	4	26.7
	Grade 2	6	40.0
	Grade 3	5	33.3
	Grade 4	0	0
Fibrosis	Present/Absent	29/1	96.7/3.3
Acellular mucin pools	Present/Absent	6/24	20.0/80.0
Infarct-like necrosis	Present/Absent	2/28	6.7/93.3
Foamy macrophages	Present/Absent	8/22	26.7/73.3
Giant cell reaction	Present/Absent	12/18	40.0/60.0
Cytosteatonecrosis	Present/Absent	6/24	20.0/80.0
Venous invasion	Present/Absent	3/27	10.0/90.0
Lymphatic invasion	Present/Absent	10/20	33.3/66.7
Perineural invasion	Present/Absent	4/26	13.3/86.7
Dystrophic calcifications	Present/Absent	9/21	30.0/70.0
Metastasized nodes	Present/Absent	4/26	13.3/86.7

Regarding the inflammatory infiltrates, in the tumor tissue, a mononuclear-type infiltrate predominated and in the adjacent healthy peritoneum, a polymorphonuclear-type infiltrate predominated, although the mononuclear type was also quite frequent.

According to the observed characteristics, PRGS and TRG values were obtained for each histological specimen and both the mean value, and the worst value were considered, that is, the highest value for each patient (Table 4).

**Table 4:** Evaluation of PRGS, TRG and the size of the peritoneal metastasis.

	Minimum	Maximum	Mean	Median	Standard deviation
Mean PRGS	1	4	1.75	1.45	0.905
Highest PRGS	1	4	2.37	2.50	1.38
Mean TRG	1	5	1.93	1.635	1.13
Highest TRG	1	5	2.7	3.5	1.66
Size of metastasis	0.50 mm	55.0 mm	10.56 mm	7.50 mm	11.29

### Outcome

For our sample, a mean overall survival value of 79.69 months was obtained, with a post-procedure survival of 56.60 months (mean) and a mean progression-free survival of 36.65 months.

### Comparison between histological findings and outcome

To compare the histological findings and the outcome – represented in the terms of overall, post-procedure and progression-free survival-, patients were divided into two groups regarding the worst PRGS value for each patient: patients with PRGS one and two, i.e. complete/major histologic response vs. PRGS 3 and 4 patients, i.e. minor histologic response or no response.

As for overall survival, we did not identify statistically significant differences in the two groups regarding PRGS.

We found that there is a statistically significant difference between the mean post-procedure survival of patients with PRGS 1 or 2 (74.19 months) and patients with PRGS 3 or 4 (25.27 months) [(log rank p=0.045), (HR 3.183, CI 95% 0.959–10.565, p=0.059)].

As for progression-free survival (PFS), the same was observed: PRGS 1 and 2 had a mean PFS of 58.03 months while in patients with PRGS 3 and 4, it was 11.67 months [(Log Rank p=0.002), (HR 4.034, CI 95% 1. 491 – 10.916, p=0.006)].

Regarding the TRG, also using the worst value obtained for each patient, the patients were divided into three groups: TRG 1–2, TRG 3 and TRG 4–5.

As for overall survival, we did not identify statistically significant differences in the three groups of TRGS.

We found that there is a statistically significant difference between the mean post-procedure survival of patients with TRG 1–2 (74.58 months), TRG 3 (17 months) and patients with TRG 4–5 (25.27 months), (log rank p=0.032), as shown in Table 5.

**Table 5:** Survival analysis – cox regression, crude HR.

	HR	Crude	p-Value
		CI 95%	
**Post-procedure survival**			
TRG 1-2	1		
TRG 3	10.150	0.942–10.150	0.056
TRG 4-5	4.224	1.113–16.030	0.034
**Progression-free survival**			
TRG 1-2	1		
TRG 3	7.078	0.061–65.817	0.085
TRG 4-5	4.843	1.672–14.034	0.004

HR, hazard ratio; CI 95%, confidence interval 95%.

As for progression-free survival, we observed that TRG 1–2 had a mean PFS of 61.68 months, TRG 3 had a mean PFS of 7 months and in patients with TRG 4–5 it was 11.67 months (log rank p=0.003), as shown in Table 5.

**Figure 2:**
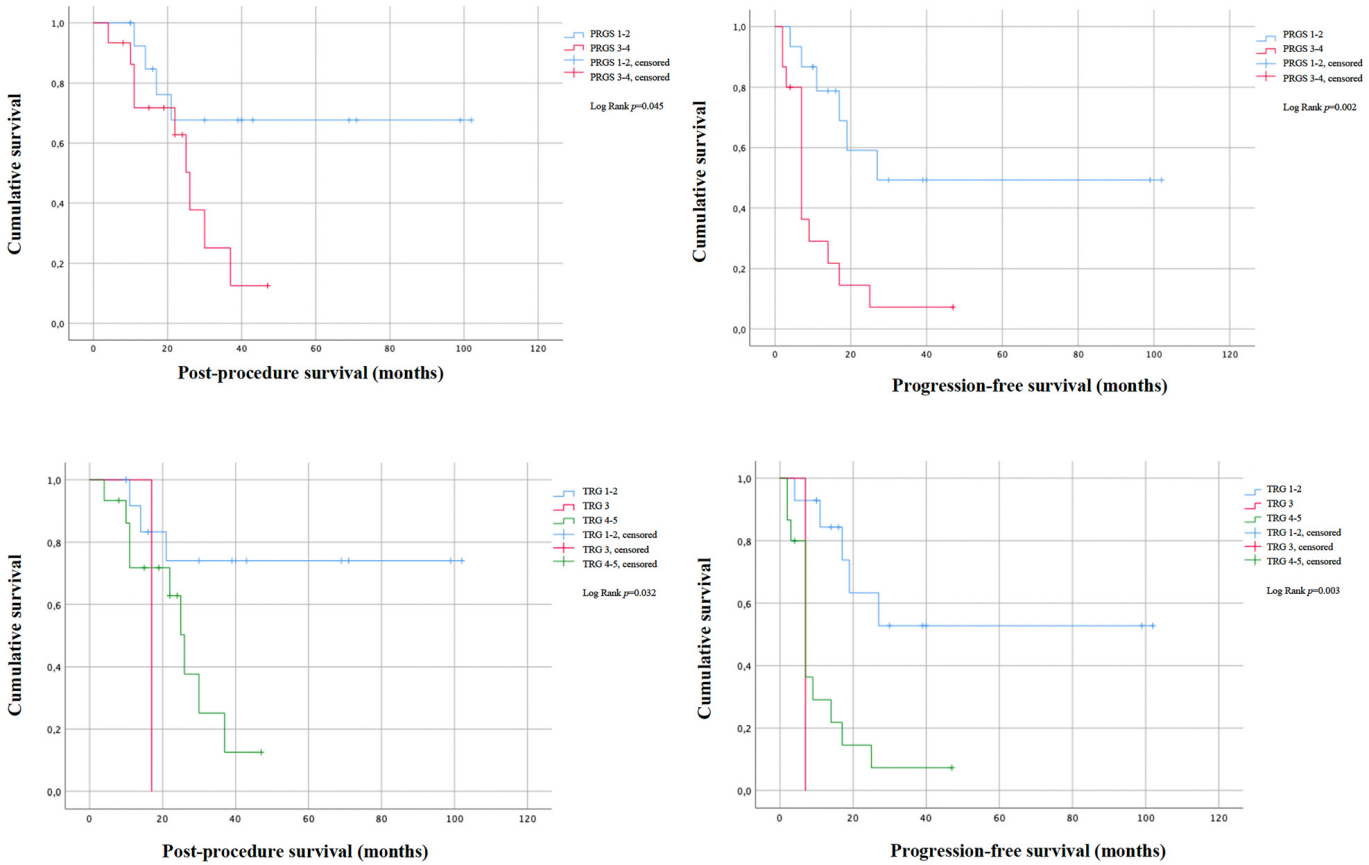
Cumulative survival curves for post-procedure survival and progression-free survival, presented in months, for each group of PRGS and TRG.

Survival curves are shown in Figure 2.

In a multivariate analysis, adjusting for the variables ECOG, stage T, PCI, completeness cytoreduction score and PRGS, it was found that PRGS remains as a prognostic factor (HR 0.249; CI 95% 0.074–0.834; p=0.024), regarding to progression-free survival.

Repeating the analysis with the TRG, keeping the remaining variables, the same was verified and TRG remained as a prognostic factor (HR 0.249; CI 95% 0.074–0.834; p=0.024).

### Comparison between preoperative chemotherapy regimens and histological findings

To determine whether there is a relationship between the different preoperative chemotherapy regimens and survival, the regimens were divided into two groups: conventional regimens and regimens that included an immunological drug.

Comparing the two types of regimens in terms of survival (overall, post-procedure and progression-free) and comparing them in terms of PRGS and TRG groups, no statistically significant differences were detected in any of the analyses.

### Evaluation of progression

To study whether there is a relationship between having a worse histological response to preoperative chemotherapy and the frequency of progression, we compared the two PRGS groups regarding progression (yes vs. no) and found that patients with PRGS 3–4 progress more than patients with PRGS 1–2, and this difference is statistically significant (86.7% vs. 40.0%, p=0.021). The odds of the PRGS 3–4 group are 9.75 times the odds of the PRGS 1–2 group to progress (p=0.014).

As for the TRG, the results were similar and we found that the TRG 4–5 group progressed more than the TRG 1–2 group, and this difference is statistically significant (86.7% vs. 35.7%, p=0.008). The odds of the TRG 4–5 group are 11.7 times the odds of the TRG 1–2 group to progress (p=0.009).

We also analyzed whether there was any kind of relationship between PRGS and TRG values and the type of progression that occurred, that is, whether patients with higher values of these scores tended to present peritoneal progression instead of extraperitoneal progression. However, we did not detect statistically significant differences between the groups.

## Discussion

This study was designed to assess the prognostic impact of histological tumor regression in patients with peritoneal carcinomatosis from colorectal cancer treated with cytoreduction surgery and HIPEC.

The histological response of peritoneal metastasis to preoperative chemotherapy was assessed through the evaluation of different representative characteristics of regression, which culminated in obtaining two different scores – PRGS and TRG – already validated in the context of digestive neoplasms. We also assessed overall survival, post-procedure survival, and progression-free survival.

Preoperative chemotherapy plays a key role in the curative strategy of these patients. The evaluation of the tumor response to this treatment remains a challenge due to the limitations inherent to imaging exams, so the histological evaluation of this response has been gaining importance. The relevance of our investigation is due to the fact that there is currently a shortage of articles that demonstrate the clinical significance of this evaluation.

The prognostic impact of the histological response of peritoneal metastasis to chemotherapy has already been demonstrated in at least two studies – a retrospective Japanese study with 142 patients and a recent French study with the same number of participants. The latter achieved a 5-year survival of 75 and 57% for complete and major response, respectively. In this same study, it was demonstrated that the histological tumor response is an independent predictor of survival [11].

The findings of our study corroborate the prognostic impact of PRGS and TRG, as patients who had a good response to preoperative chemotherapy (PRGS 1–2 and TRG 1–2) had substantially higher post-procedure and progression-free survival compared with those patients who had a poor response, i.e., poor tumor regression. That is, we were also able to demonstrate that these two scores are predictors of survival. Our study also reinforced the idea that it is possible to obtain a complete tumor response and the curative potential of this treatment, which considerably prolonged the survival of these patients.

One of the challenges we faced was to distinguish regression findings from so-called normal findings, unrelated to treatment response. Typically, peritoneal metastasis not treated with chemotherapy show large areas of viable tumor cells, accompanied by areas of necrosis, without fibrosis. On the other hand, tumor regression is characterized by histological reorganization, with a decrease in the number or total disappearance of viable tumor cells, which are replaced by fibrosis. Fibrosis is a primary indicator of response to chemotherapy, being one of the characteristics considered in the assessment of histological response.

We also know that necrosis in response to chemotherapy is mainly infarct-like necrosis, which is defined as confluent areas of eosinophilic staining cellular remnants, surrounded by fibrosis and often with other associated findings such as xanthelasmized macrophages, microcalcifications and clefts of cholesterol. This differs from “dirty” necrosis, not considered a regression feature, in which we see irregularly dispersed nuclear debris. It happens that, sometimes, infarct-like necrosis can also be seen in the center of untreated lesions, in response to cellular hypoxia [1].

Another regression finding is the pools of acellular mucins. These differ from the cellular mucins that are frequent in these tumors since they are often mucinous. So, the latter are not considered a regression feature.

In order to try to overcome this difficulty, the so-called “healthy” peritoneum that is adjacent to the peritoneal metastasis was also evaluated. This allowed us to make a better distinction between regression findings and normal findings.

Another difficulty we faced was that the slides were exclusively stained with hematoxylin-eosin, which made it difficult to assess some characteristics such as vascular invasion. However, this staining is the one recommended in the PRGS assessment protocols, although additional stains may be useful.

One of the major limitations of our study was the small sample size. This justifies the discordant findings obtained in the group TRG 3, since this group consisted of only one patient, which meant that the values obtained for this group were not significant. However, this sample size reflects the fact that this treatment is applied to carefully selected patients and is a complex treatment, which requires an experienced and multidisciplinary team.

During surgery, multiple fragments are removed from different areas, from which multiple slides are obtained. Within these we often observed different degrees of regression. There is still no consensus as to whether we should consider only the worst degree obtained, that is, the highest value of PRGS or TRG, or the mean value. Thus, we chose to record both values in this investigation, although we focused more on the higher value, as it seemed more appropriate to the context of our sample.

This variability of regression between samples from the same patient remains to be clarified, which may be due on one hand to biological factors such as different tumor clones or due to therapy, as for example in the case of cell cycle-dependent cytostatics or by the heterogeneity of drug distribution.

One aspect that may also have had an impact is the heterogeneity of chemotherapy regimens. However, we were unable to demonstrate any kind of significant difference. Studies with a larger sample and with a greater variety of regimens performed may obtain significant findings regarding this topic.

It is important that, in the future, further studies are carried out to assess the prognostic impact of PRGS and TRG, with larger samples, multicentric and possibly prospective studies, to try to overcome some of the limitations we faced.

Metastatic colorectal cancer remains a condition with a major impact on patient survival. However, preoperative chemotherapy followed by cytoreduction surgery and hyperthermic intraperitoneal chemotherapy in selected patients may prolong survival for about 57 months, as demonstrated in our investigation.

The histological findings that typically represent a response to therapy are fibrosis, xanthelasmized macrophages, infarct-like necrosis, acellular mucin pools and, above all, the absence of viable tumor cells. This response can be represented by scores such as the peritoneal regression grading score and the tumor regression grading.

A better histological response to preoperative chemotherapy, represented as a lower PRGS and TRG value, is associated with longer post-procedure survival and progression-free survival in this group of patients.

Thus, these two scores have prognostic value and possibly can overcome the limitations inherent to imaging tests and allow for more appropriate and individualized therapeutic decisions.
